# Targeting Amyloidogenic Processing of APP in Alzheimer’s Disease

**DOI:** 10.3389/fnmol.2020.00137

**Published:** 2020-08-04

**Authors:** Jing Zhao, Xinyue Liu, Weiming Xia, Yingkai Zhang, Chunyu Wang

**Affiliations:** ^1^Center for Biotechnology and Interdisciplinary Studies, Rensselaer Polytechnic Institute, Troy, NY, United States; ^2^Geriatric Research Education Clinical Center, Edith Nourse Rogers Memorial Veterans Hospital, Bedford, MA, United States; ^3^Department of Pharmacology and Experimental Therapeutics, School of Medicine, Boston University, Boston, MA, United States; ^4^Department of Chemistry, New York University, New York, NY, United States; ^5^Department of Biological Sciences, Rensselaer Polytechnic Institute, Troy, NY, United States; ^6^Department of Chemistry and Chemical Biology, Rensselaer Polytechnic Institute, Troy, NY, United States

**Keywords:** Alzheimer’s disease, amyloid-β, β-secretase inhibitor, γ-secretase inhibitors, γ-secretase modulator, clinical trial

## Abstract

Alzheimer’s disease (AD) is the most common type of senile dementia, characterized by neurofibrillary tangle and amyloid plaque in brain pathology. Major efforts in AD drug were devoted to the interference with the production and accumulation of amyloid-β peptide (Aβ), which plays a causal role in the pathogenesis of AD. Aβ is generated from amyloid precursor protein (APP), by consecutive cleavage by β-secretase and γ-secretase. Therefore, β-secretase and γ-secretase inhibition have been the focus for AD drug discovery efforts for amyloid reduction. Here, we review β-secretase inhibitors and γ-secretase inhibitors/modulators, and their efficacies in clinical trials. In addition, we discussed the novel concept of specifically targeting the γ-secretase substrate APP. Targeting amyloidogenic processing of APP is still a fundamentally sound strategy to develop disease-modifying AD therapies and recent advance in γ-secretase/APP complex structure provides new opportunities in designing selective inhibitors/modulators for AD.

## Introduction

Alzheimer’s disease (AD) is a progressive and incurable neurodegenerative disorder, characterized by progressive and irreversible loss of memory. AD is the leading cause of senile dementia. The number of people age 65 and older living with AD’s dementia in the United States is projected to grow from 5.8 million in 2020 to 13.8 million by 2050 (Alzheimer’s_Association, 2020). Cognitive deficits caused by AD, such as progressive memory loss, difficulty in communication and movement disorder, significantly compromise the patients’ quality of life, leading to hospitalization and eventually death due to complications. AD has been recognized as one of the most difficult medical problems with hefty economic burden (Wimo et al., 2010). Total medical expenses for Alzheimer’s or other dementias in the United States are projected to be $305 billion in 2020 (Alzheimer’s_Association, 2020). The cost of AD is likely to skyrocket in the near future, due to rising ageing population, increasing mortality relative to other disease and the absence of a disease-modifying drug. Therefore, there is a major unmet medical need for disease-modifying therapies for AD.

Approved drugs for AD include acetylcholinesterase inhibitors (Aricept^®^, Exelon^®^, Razadyne^®^) and NMDA-antagonist memantine (Namenda^®^). However, these drugs are not disease-modifying, only improving symptoms without slowing down or stopping AD from progressing. In the last 30 years, a large number of drug candidates have entered clinical development but no new drug for AD has been approved since memantine in 2003. The majority of AD drug discovery focused on inhibiting the amyloid-β peptide (Aβ) production from the amyloidogenic processing of APP.

## Amyloid Cascade Hypothesis and Amyloidogenic Processing of APP

In the past 30 years, the amyloid hypothesis has been extensively tested and amyloid has been the most compelling therapeutic target for AD. Despite ongoing debates about this hypothesis in light of recent failures of anti-amyloid-based clinical trials, new evidences continue to emerge to support the idea that an imbalance between production and clearance of Aβ peptides is the initiating event of AD pathogenic processes. Abnormal accumulation of amyloid eventually leads to formation of senile plaques and neurofibrillary tangles, two pathological hallmarks of AD. Aβ aggregates were found to be toxic both *in vitro* and *in vivo.* Numerous studies have shown that Aβ aggregates, especially soluble oligomers, impair both synaptic function and structure (Kokubo et al., 2005; Wilcox et al., 2011). Injection of soluble Aβ42 oligomers directly isolated form AD cerebral cortex into healthy rats leads to impaired memory (Selkoe et al., 2016). In addition, accumulation of Aβ oligomers can not only trigger AD-type tau hyperphosphorylation and cause neurotic dystrophy (Jin et al., 2011; Stancu et al., 2014; Jacobs et al., 2018), but also activate neuroinflammation (Park et al., 2018; Henstridge et al., 2019). Apolipoprotein E4, the greatest genetic risk factor for late-onset AD, impairs Aβ clearance and promotes Aβ accumulation in the brain (Carter et al., 2001; Wildsmith et al., 2013). Along with tau, Aβ might be transmissible through the Aβ contaminants in cadaver-derived human growth hormone for the treatment of Creutzfeldt-Jakob disease (Duyckaerts et al., 2018). From human genetics, dominant mutations causing early-onset familial AD reside either in APP or presenilin (catalytic sub-unit of γ-secretase), which alter the proteolytic processing of APP in ways either elevating the Aβ_42_/Aβ_40_ ratio or increasing the self-aggregation propensity of resultant Aβ peptides (De Jonghe, 2001; Selkoe, 2001; Chen et al., 2014). Duplication of the APP gene in Down’s syndrome leads to Aβ deposits in the teens, and almost invariably leads to AD at an early age (Lejeune et al., 1959; Head et al., 2012). Interestingly, three DS patients with partial trisomy that excludes the APP gene did not develop dementia (Korbel et al., 2009; Doran et al., 2016). The human genetics of DS strikingly demonstrates that increasing Aβ dosage (APP duplication) causes dementia, while normalized Aβ dosage in DS (partial trisomy without APP duplication) prevents dementia (albeit the sample size = 3 is low), affirming that amyloid reduction is a fundamentally sound strategy for disease-modifying treatment of AD. The failures of anti-amyloid clinical trials in recent years can be attributed to giving the therapy too late to the patients, poor clinical trial design, heterogeneity of the trial patient population etc.

**FIGURE 1:**
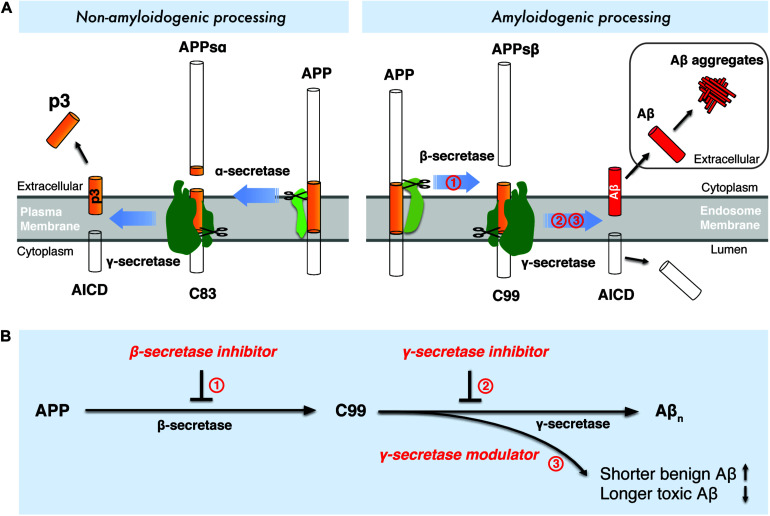
Drugdiscovery strategies targeting the amyloidogenic processing of APP. **(A)** Amyloidogenic and non-amyloidogenic processing of APP. **(B)** Drug discovery strategies targeting β-secretase and γ-secretase.

Aβ is a small peptide generated by proteolytic processing of APP (Figure 1A), a type-I transmembrane protein with a large extracellular domain. APP is transported to the plasma membrane through the endoplasmic reticulum -Golgi secretory pathway. The majority of APP is processed via the non-amyloidogenic pathway at the plasma membrane (Figure 1A). In the non-amyloidogenic pathway, APP is cleaved by α-secretase within the Aβ domain between Lys16 and Leu17, producing a soluble N-terminal fragment (APPs α) and a membrane-bound C-terminal fragment, C83, which can be further cleaved by γ-secretases and generates a soluble extracellular p3 peptide, thus precluding the formation of intact Aβ (Figure 1A; Anderson et al., 1991; Sisodia, 1992; Wilson et al., 1999). Unlike the non-amyloidogenic pathway, APP is internalized and delivered to endosomes in the amyloidogenic pathway (Bu, 2009). During amyloidogenic APP processing, APP is cleaved by β-secretase (BACE1, β-site APP-cleaving enzyme 1), generating a soluble N-terminal fragment (APPsβ) and a membrane-bound C-terminal fragment (C99) (Vassar, 2004; Vassar et al., 1999). Within the membrane, C99 is subsequently cleaved by an enzymatic complex known as γ-secretase, releasing a cytoplasmic polypeptide termed AICD (APP intracellular domain) at the luminal side and Aβ peptides (Thinakaran and Koo, 2008) at the other side of the membrane. AICD is transferred to the nucleus, where it functions as a transcriptional factor (Berridge, 2010), whereas the Aβ peptides are secreted into the extracellular space when the endosome recycles to cell surface. γ-Secretase cleaves APP at variable sites within the transmembrane domain, generating Aβ peptides ranging in length from 38 to 43 residues (Selkoe and Wolfe, 2007). Among different Aβ species, Aβ_42_ and Aβ_43_ are highly self-aggregating, while Aβ_40_ and shorter peptides are relatively benign (Burdick et al., 1992). Aβ_42_ and Aβ_40_ are the two common Aβ species in the human brain and the increased Aβ_42_/Aβ_40_ ratio is a common biochemical feature in the early-onset familial AD (FAD) caused by mutations in APP and presenilin. Aβ_42_ aggregates rapidly into neurotoxic oligomers, leading to fibrils and plaques. It has been proposed that Aβ oligomers are more toxic than fibrils, therefore it may play a more important role than amyloid plaque in AD progression. Aberrant process of APP by β-secretase and γ-secretase may result in imbalance between production and clearance of Aβ peptides, leading to toxic oligomers, fibrils and senile plaques. Interestingly, pathogenic mutations in presenilin were found to destabilize γ-secretase-APP interactions and thus enhance the production of longer Aβ peptides (Chévez-Gutiérrez et al., 2012; Veugelen et al., 2016; Szaruga et al., 2017). These finding points to enhancing the stability of γ-secretase-Aβ_n_ complex as a potential therapeutic approach for AD.

## Drug Discovery Targeting Amyloidogenic Processing of APP

Interference with the amyloidogenic processing of APP has been a major strategy to modulate Aβ production. As two crucial enzymes catalyzing the intramembrane proteolysis of APP, β-secretase and γ-secretase have been the most prominent targets for AD drug discovery. In the past two decades, numerous β-secretase inhibitors and γ-secretase inhibitors/modulators were discovered to inhibit or modulate the amyloidogenic processing of APP, either causing the reduced production of total Aβ_*n*_ or shifting the production of Aβ to shorter and more benign Aβ species (Figure 1B).

### Drug Discovery Targeting β-Secretase

#### β-Secretase

β-secretase, also named as BACE1 (β-site APP-cleaving enzyme 1), was identified in 1999 (Fairbanks et al., 1999; Hussain et al., 1999; Vassar et al., 1999; Yan et al., 1999; Lin et al., 2000) as an APP-cleaving aspartyl protease. BACE1 is the principal neuronal protease for generating C99 from APP, which leads to subsequent Aβ generation by γ-secretase. Importantly, BACE1 shedding of APP is a prerequisite for γ-secretase cleavage within the transmembrane domain of APP for Aβ production. Secretion of Aβ peptides is abolished in cultures of BACE1-deficient embryonic cortical neurons (Cai et al., 2001). Naturally, BACE1 inhibition has been a widely pursued therapeutic target for amyloid reduction.

**FIGURE 2:**
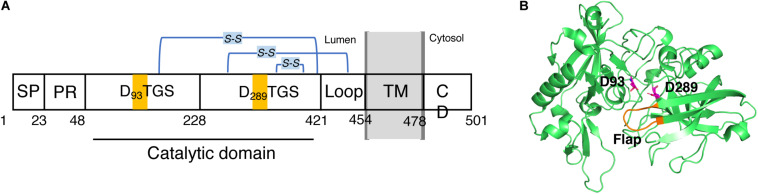
Schematic view of the domains of BACE1 and structure of its catalytic domain. **(A)** BACE1 is a membrane protein consists of (from N- to C- terminal) a signal peptide (SP, 1–22), a proline rich domain (PR, 23–47), a large luminal catalytic domain (48-420), a loop (421-453), a transmembrane domain (TM, 454-477), and a cytoplasmic domain (CD, 478-501). **(B)** 3D structure of the catalytic domain. Two conserved catalytic aspartic acids, D93 and D289, are highlighted.

BACE1 is a type-I membrane protein with 501 amino acid residues related to the pepsin family. It is localized within acidic subcellular compartments of the secretory pathway, primarily the Golgi apparatus and endosomes. As shown in Figure 2A. BACE1 has an N-terminal signal sequence (residues 1–21), a pro-peptide domain (residues 22–45), a large luminal catalytic domain (residues 46-451), a single transmembrane domain (residues 452-483), and a short cytoplasmic domain (residues 484-501) (Hussain et al., 1999; Benjannet et al., 2001). In addition, BACE1 has several N-linked glycosylation sites and six cysteine residues that form three intramolecular disulfide bonds, C216-C420, C278-C443, and C330-C380. The N-terminal signal sequence and pro-peptide domain were removed post-translationally, so the mature BACE1 sequence begins at residue Glu46 (Creemers et al., 2001). In the luminal catalytic domain for the β-site cleavage of APP, BACE1 contains two motifs, DTGS (residues 93–96) and DSGT (residues 289–292), which contain the two highly conserved catalytic aspartates (Hussain et al., 1999; Bennett et al., 2000). The crystal structure of the catalytic domain (residues 56-446) of BACE1 with an inhibitor was first published by Hong et al. in 2000 (PDB code: 1FKN) (Hong et al., 2000). Like other aspartic proteases, the substrate binding cleft was located between the N- and C-terminal lobes of BACE1, together with a β-hairpin loop which forms the flap region. The flap opens to allow the substrate to enter and then closes down on the substrate during catalysis and reopens to release the hydrolyzed products. As shown in Figure 2B, the two conserved aspartate, D93 and D289, are located at the groove between the N- and C-terminal lobes, partially covered by the “flap” (residues 130-135) (Hong et al., 2000). A later substrate-free (apo) structure of BACE1 (PDB code: 1W50) showed a water molecule located between D93 and D289, which is likely involved in nucleophilic attack for peptide hydrolysis and important binding site for inhibitors (Patel et al., 2004; Ghosh and Osswald, 2014).

BACE1 cleavage is highly specific and cleaves APP only at the β-secretase sites of Asp1 and Glu11 of Aβ (Vassar et al., 1999). As expected, BACE1 cleaves the Swedish APP mutant 10 to 100-fold more efficiently than wild-type APP. BACE1 appeared to be an ideal target for drug discovery since its complete inhibition should shut down the amyloidogenic APP processing.

#### Drug Discovery Targeting BACE1

**FIGURE 3:**
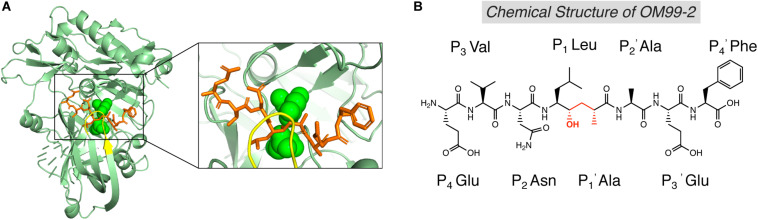
Structure of BACE1 complexed with peptidomimetic inhibitor OM99-2. **(A)** Crystal structure of BACE1-OM99-2 complex (PDB code: 1FKN). The ligand binding region on BACE1 was magnified on the right. Inhibitor OM99-2 was shown as sticks in orange. Two catalytic aspartic acid residues were shown in green as sphere. The “flap” region was shown in yellow. **(B)** Chemical structure of OM99-2. The hydroxyethylene transition-state isostere was indicated in red.

The first generation of BACE1 inhibitors are peptidomimetic transition-state analogs, which were designed to be accommodated within the substrate-binding cleft of BACE1. In 2000, Hong et al. developed an 8-residue peptide OM99-2 utilizing the template of the β-cleavage site of Swedish APP, where the scissile peptide bond was replaced by a Leu-Ala hydroxyethylene transition-state isostere (Figure 3B; Ghosh et al., 2000). The crystal structure of BACE1 and OM99-2 complex (Figure 3A) provided coveted molecular insight into the ligand/enzyme interaction and significantly advanced the development of BACE1 inhibitors (Hong et al., 2000). Catalytic aspartates D93 and D289 (see Figure 3A, in green) are located at the center of the substrate binding pocket. There are four hydrogen bonds between the catalytic aspartates and the transition state isostere hydroxyl, and ten hydrogen bonds between the binding cleft and flap to OM99-2 backbone. As shown in Figure 3A, the “flap” (in yellow) closes over the top of the cleft upon the binding of the inhibitor. Futher investigation led to the design of inhibitor OM00-3 by the replacement of the P2’ Ala in OM99-2 with a Val, which achived a five-fold enhancement of inhibitory potency (Hong et al., 2002). Additional peptidomimetic inhibitors were developed, including KMI-008, KMI-420, and KMI-429 by Kiso’s group (Shuto et al., 2003; Kimura et al., 2005; Asai et al., 2006), GSK188909 (Hussain et al., 2007), and compound 11 (Iserloh et al., 2008), etc. However, the big molecular size precludes the application of peptidomimetic BACE1 inhibitors *in vivo*, due to their short half-life, deficiency in crossing the BBB, and low oral availability. Therefore, later generations of BACE1 inhibitors are mostly non-peptidic small molecules.

**FIGURE 4:**
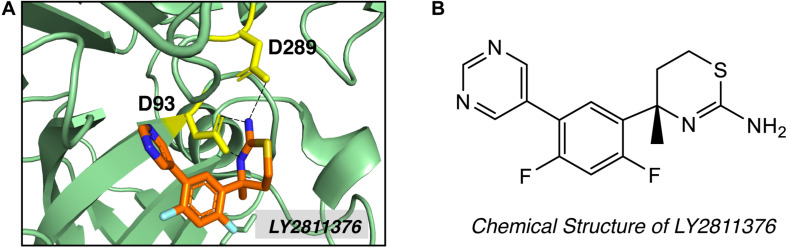
Structure of BACE1 complexed with small molecue inhibitor LY2811376. **(A)** LY2811376 binds to the active site of BACE1 (PDB code: 4YBI). Inhibitor LY2811376 was shown as sticks colored with orange for carbon, blue for nitrogen, light blue for fluorine and light-orange for sulfur. The side chain of two catalytic aspartate (D93 and D289) were shown in yellow as sticks. Hydrogen bonds between aspartate side chain and LY2811376 were shown in dotted black lines. **(B)** Chemical structure of LY2811376.

High-throughput screening (HTS) and fragment-based drug discovery (FBDD) are two major methods employed to explore non-peptidyl small molecule BACE1 inhibitors. In contrast to traditional HTS, which uses libraries of drug-like compounds (typically 300–500 Da), FBDD screens libraries of small fragments (typically < 300 Da), allowing more thorough exploration of the chemical space and the identification of weakly binding chemical fragments. Small fragment hits can then be linked or extended into unique multi-fragment scaffold (Erlanson, 2012). Screening of 8000 fragments by Lilly Research Laboratories produced two promising fragment hits, amino-benzothiazine and amino-thiadiazine (May et al., 2011). Evolution of the fragment based on crystal structure produced compound LY2811376 [(S)-4-(2,4-difluoro-5-pyrimidin-5-yl-phenyl)-4-methyl-5,6-dihydro- 4H-[1,3]thiazin-2-ylamine]. LY2811376 was the first orally available, non-peptidic small-molecule BACE1 inhibitor, showing satisfactory pharmacokinetic and pharmacodynamic properties in preclinical animal models and in humans (May et al., 2011). The chemical structure of LY2811376 and its complex with BACE1 were shown in Figure 4. LY2811376 binds to the active site of BACE1 and forms an optimal H-bonds network with the catalytic aspartates (May et al., 2011). Despite the encouraging results in preclinical animal models, LY2811376 shows toxicity in long-term studies. Other fragment-based compounds, including AZD3839 from Astra-Zeneca (Jeppsson et al., 2012) and NB-360 from Novartis (Neumann et al., 2015), also showed great potential in reducing brain Aβ levels in preclinical studies.

#### BACE1 Inhibitors in Clinical Trials

BACE1 inhibitors effectively reduced brain and CSF Aβ levels in both animal studies and human clinical trials. In the past two decades, many potent BACE inhibitors have been developed, but only a small portion entered the clinical trials. Selectivity over other aspartic protease (BACE2, pepsin, renin, cathepsin D, and cathepsin E) and blood-brain barrier (BBB) permeability are major hurdles. The first Phase I clinical trial on a BACE1 inhibitor, CTS-21166, was conducted by CoMentis in 2008. CTS-21166 passes BBB, with high oral bio-availability and selectivity of BACE1 over other proteases. Results from clinical studies indicated a dose-dependent reduction of plasma Aβ for an extended period of time, with up to 80% inhibition at the highest dosage (Gabrielle, 2008). Merck conducted phase I clinical trial on their inhibitor MK-8931 (Verubecestat) in 2012, which is well-tolerated and demonstrates a profound (up to 94%) reduction in CSF Aβ (Forman et al., 2012). MK-8931 is the first BACE1 inhibitor to advance to phase II/III clinical trial in patients with mild to moderate AD. It was discontinued in 2017 (Table 1), due to a lack of clinical benefit in cognition (Egan et al., 2018). Subsequent phase III clinical trials of MK-8931 in patients with prodromal AD was also discontinued in 2018, because it was deemed unlikely to exhibit a positive benefit/risk ratio (Merck, 2018). Several other promising BACE1 inhibitors in late state clinical trials also reported disappointing results, including LY2886721 (Eli Lilly), AZD3839 (AstraZeneca), atabecestat (JNJ-54,861,911, Janssen), and lanabecestat (AZD3293, LY3314814, AstraZeneca and Eli Lilly) (Table 1).

**TABLE 1:**
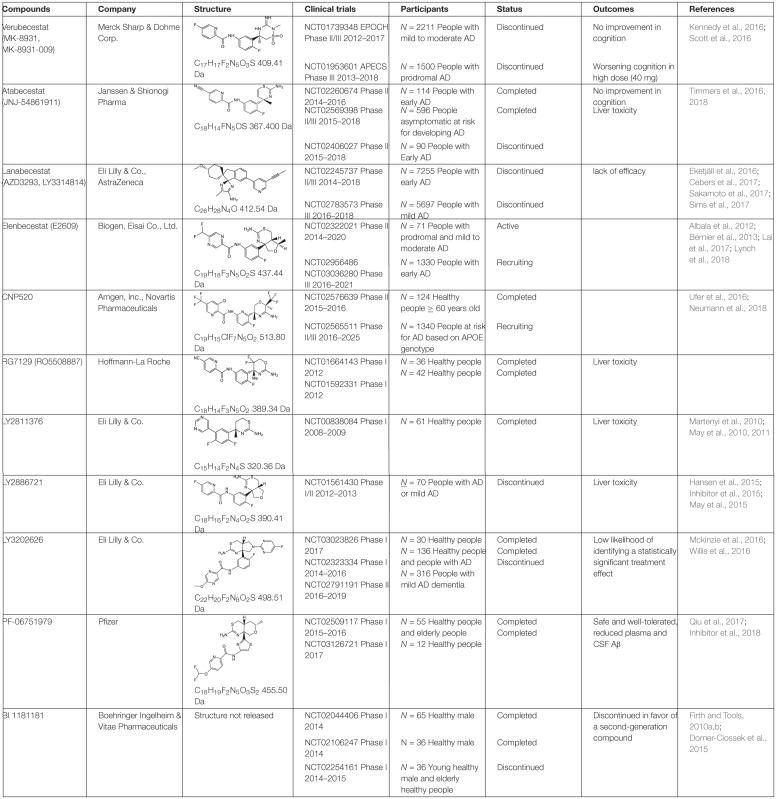
BACE1 inhibitors in clinical trials.

### Drug Discovery Targeting γ-Secretase

#### γ-Secretase

**FIGURE 5:**
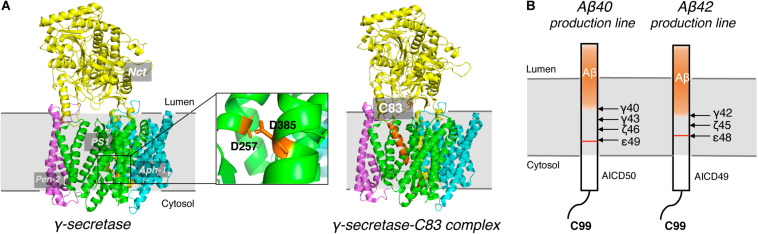
Cryo-EM Structure of γ-secretase and progressive cleavage of APP transmembrane domain. **(A)** The cryo-EM structure of γ-secretase apo (left) and complexed with substrate C83 (right). Four subunits, Nct, PS1, Pen-2 and Aph-1, were indicated. Two conserved catalytic aspartates, D257 and D385, were shown as sticks in orange in the magnified graph. The APP substrate C83 was shown as cartoon in orange in the complex. **(B)** Schematic diagram of the progressive cleavage sites of γ-secretase on substrate C99.

γ-secretase is a membrane protein complex composed of four essential subunits, with presenilin-1 (PS1) or presenilin-2 (PS2) as the catalytic subunit, nicastrin (Nct), anterior pharynx-defective 1 (Aph-1), and presenilin enhancer 2 (Pen-2) (De Strooper, 2003; Wolfe and Kopan, 2004; Wolfe, 2006). PS1 undergoes autocleavage through its endopeptidase activity to produce a N-terminal fragment (NTF) and a C-terminal fragment (CTF) (Thinakaran et al., 1996), each of which contributes a conserved catalytic aspartate, D257 and D385, respectively (Figure 5A). Nct is believed to play a role in substrate recognition and its initial docking (Shah et al., 2005). Aph-1 stabilizes the complex and Pen-2 is required for maturation of γ-secretase (Takasugi et al., 2003). γ-secretase cleaves type I transmembrane proteins and has more than 90 reported substrates (Haapasalo and Kovacs, 2011; Jurisch-Yaksi et al., 2013), of which APP and Notch are the most well-characterized. After the cleavage of APP by BACE1, a C-terminal 99-residue fragment of APP (C99) is generated and subsequently cleaved by γ-secretase. The initial cleavage of γ-secretase produces a 48-residue (Aβ48) or 49-redidue (Aβ49) amyloid peptide, while at the same time the intracellular domains (AICD) are liberated into the cytoplasm (Figure 5B; De Strooper et al., 1998; Wolfe et al., 1999). Subsequent cleavage of Aβ49 by the C-terminal peptidase activity of γ-secretase leads to the generation of Aβ46, Aβ43, and Aβ40, while cleavage of Aβ48 results in Aβ45, Aβ42, and Aβ38 (Golde et al., 1992; Sano et al., 2009). Among Aβ peptides with different lengths, Aβ_42_ and Aβ_43_ are most prone to aggregation, while Aβ_40_ and shorter peptides are relatively benign (Burdick et al., 1992).

#### Drug Discovery Targeting γ-Secretase

γ-Secretase cleaves within APP transmembrane domain (APPTM) of C99 to release Aβ, which aggregates to form neurotoxic oligomers and fibrils. Thus, γ-secretase is an obvious drug target for reducing amyloid load. Efforts have been devoted to develop γ-secretase targeting compounds, γ-secretase inhibitors (GSIs) and later γ-secretase modulators (GSMs).

**FIGURE 6:**
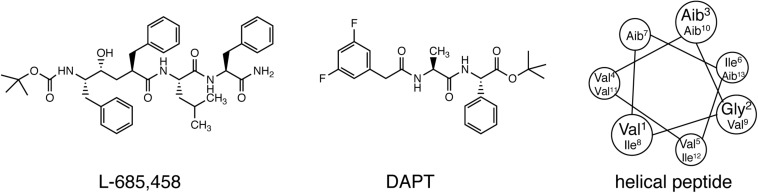
Chemical structures of representative compounds in three classes of substrate-based GSIs. From left to right: transition-state analog inhibitor (L-685,458), chemically modified compounds based on hits from high-throughput screening (DAPT), and helical peptide.

**FIGURE 7:**
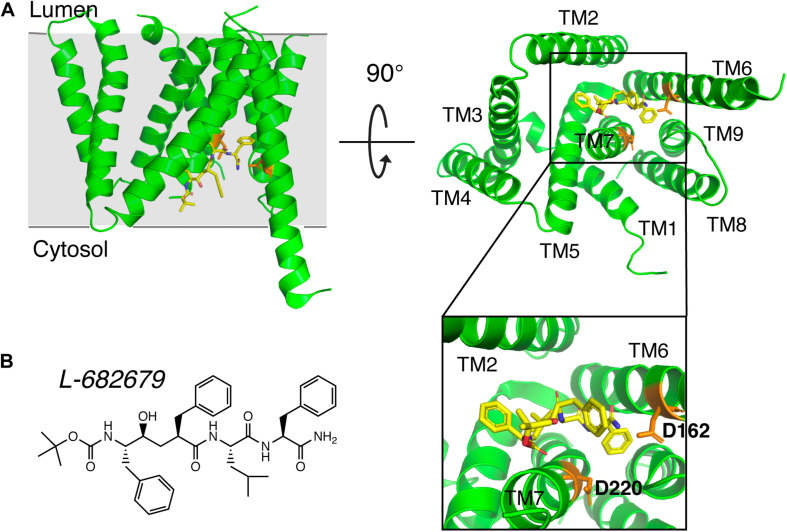
The complex structure of presenilin homolog PSH bound to a hydroxyethylene derivative L-682679. **(A)** Structure of PSH bound to L-682679. The two catalytic aspartates D162 and D220 were shown in orange. L-682679 was shown in stick model with yellow for carbon, blue for nitrogen, red for oxygen (Dang et al., 2015). **(B)** Chemical structure of L-682679.

Early studies on γ-secretase targeting compounds have shown an increase of the levels of substrates C99 and C83, and a reduction of the levels of the γ-secretase cleavage products Aβ and p3 in APP-transfected cells (Higaki et al., 1995, 1999; Klafki et al., 1996). Those compounds were named as GSIs, among which dipeptide aldehydes (such as MG-132 and MDL-28170) were firstly reported (Higaki et al., 1995; Klafki et al., 1996). At the same time, substrate-based GSIs were designed, and these compounds show pharmacological effects through occupying the binding site of APP on γ-secretase. The substrate-based GSIs (Figure 6) includes: (1) transition-state analog inhibitor of aspartyl proteases, such as hydroxyethylene L-685,458, which was identified to target PS1 NTF and CTF (Shearman et al., 2000); (2) helical peptide (Das et al., 2003), which was found to directly bind to the PS1 NTF/CTF interface (Kornilova et al., 2005); (3) Chemically modified compounds based on hits from high-throughput screening, such as DAPT (Dovey et al., 2001), for which PS1 was identified as the direct target (Morohashi et al., 2006; Bai et al., 2015). The complex structure of presenilin homolog PSH bound to a hydroxyethylene derivative L-682,679 was shown in Figure 7. PSH is composed of 9 transmembrane helices (TMs). L-682,679 was found to bind in the cleft surrounded by TMs 2, 6, 7, 8, and 9. The two catalytic aspartates D162 and D220 were separated by the phenol group in the amide end of L-682679 (Dang et al., 2015).

Additional non-peptidyl GSIs were developed, e.g., modification of DAPT led to a much more potent compound LY-411,575, which was further modified to be LY-450,139 (semagacestat, Table 2), a compound that was advanced to phase III clinical trials. However, the phase III clinical trial revealed severe gastrointestinal toxicity and skin cancer, which were probably due to impairment of Notch signaling.

**TABLE 2:**
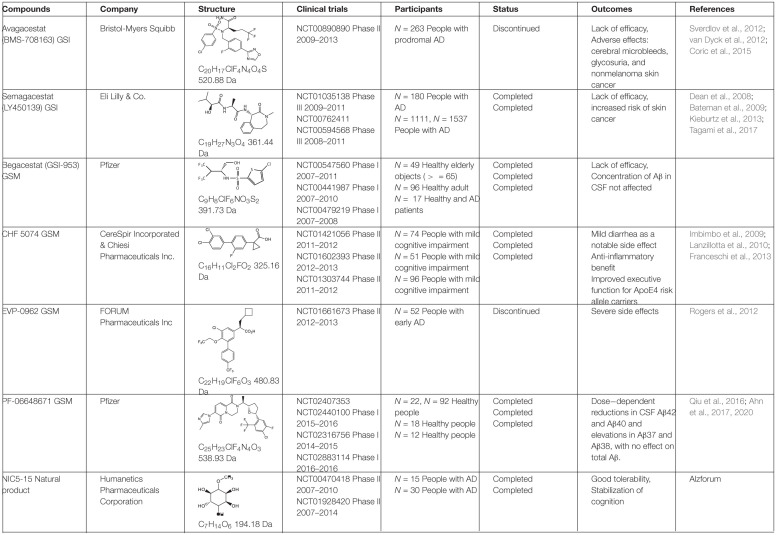
γ-secretase inhibitors/modulators in clinical trials.

To avoid the toxicity of GSIs, extensive efforts have been devoted toward selective γ-secretase inhibitors/γ-secretase modulators (GSMs) (Weggen et al., 2001). Some GSMs interact with γ-secretase through the allosteric binding site, and therefore do not interfere with the normal γ-secretase processing of other physiological substrates, such as Notch (called “Notch-sparing GSMs”). The first generation of GSMs is a subset of non-steroidal anti-inflammatory agents (NSAIDs), including ibuprofen, sulindac and indomethacin. These NSAIDs interact with PS at the luminal side and increase the distance between the N- and C- termini of PS, thus direct the cleavage sites toward Aβ38 instead of Aβ42, without altering total Aβ production (Lleó et al., 2004; Takeo et al., 2014). The second generation of GSMs include NSAID-derived carboxylic acid analogs (Beher et al., 2004; Hall et al., 2010) and non-NSAID GSMs (Huang et al., 2012; Sun et al., 2012). The second-generation GSMs have better pharmacokinetic properties, and several of them entered clinical trials, including CHF 5074, EVP-0962, and PF-06648671 (Table 2). Natural products were also screened and characterized for their GSM activities, such as dihydroergocristine (DHEC) (Lei et al., 2015), curcumin (Goozee et al., 2016), and luteolin (Wang et al., 2016). However, the mechanism of how GSMs shift Aβ production is poorly understood, partially because the complexity of the structure of γ-secretase. γ-Secretase contains 4 subunits and at least 18 transmembrane domains, structural information on this high molecular weight complex is limited until recently. Moreover, the binding sites of GSMs are still not understood. GSMs fenofibrate and tarenflurbil were initially reported to modulate γ-secretase cleavage by binding to the substrate APP (Kukar et al., 2008), but the specificity of this binding is questionable (Beel et al., 2009). Other GSMs have been reported to bind presenilin (Crump et al., 2011; Ohki et al., 2011).

#### γ-Secretase Inhibitors/Modulators in Clinical Trials

The most potent GSI semagacestat (LY450139) failed in Phase III clinical trials with more than 2600 patients from 31 countries, due to worsening cognition and increased risk of skin cancer in test patients comparing to placebo group (Kieburtz et al., 2013; Tagami et al., 2017). The side effects are likely due to the impaired Notch signaling. Focus was then shifted toward “Notch-sparing” GSMs, which shift the Aβ production to shorter and benign peptides without affecting the total Aβ production. However, avagacestat (BMS-708163), which was claimed to be a Notch-sparing GSI, also failed in a Phase II clinical trial (Table 2) with similar side effects to those found with semagacestat (Sverdlov et al., 2012; van Dyck et al., 2012; Coric et al., 2015). Later a lack of Notch selectivity was reported for avagacestat, which may contribute to the failure of clinical trial (Crump et al., 2012). Two more GSMs entered clinical trials. EVP-0962, entered phase II clinical trial in 2012 but no results have ever been reported. NIC5-15, a naturally occurring sugar alcohol found in plants and fruits, also entered phase II clinical trial without follow-up studies.

## Discussion

All clinical trials of BACE1 inhibitors and GSIs/GSMs failed, raising the question about causal role of Aβ in AD pathogenesis, and the validity of amyloid cascade hypothesis in general. However, the amyloid hypothesis refers to a continuum of pathological processes at different stages of disease lasting 15–20 years before symptoms appear (Rowe et al., 2010). It is widely accepted that amyloid biomarkers (detected by reduced Aβ42 in cerebrospinal fluid (CSF) and/or positive brain amyloid PET imaging) represent the earliest evidence of AD neuropathologic change currently detectable in humans (Donohue et al., 2014; Young et al., 2014; Xiong et al., 2016). A causative role of Aβ in AD pathogenesis is supported by strong evidences, including genetic of FAD (De Jonghe, 2001; Selkoe, 2001; Chen et al., 2014) and Down’s syndrome (Lejeune et al., 1959; Head et al., 2012), toxicity of Aβ aggregates (Selkoe et al., 2016), Aβ activation of neuron inflammation (Eng et al., 2004) and Aβ potentiation of tau pathology (Jin et al., 2011). Very recently, increased Aβ42/40 ratio was shown to drive tau pathology in 3-dimensional human AD neural cell culture models (Kwak et al., 2020). These studies support the causal upstream role for Aβ in the pathogenesis of AD.

Reducing Aβ42 by modulating β- or γ-secretase activity may inhibit subsequent neurodegenerative changes in the brain. It is clear that Aβ alone is not sufficient for cognitive dysfunction, but it may play a crucial role in potentiating downstream tauopathy and neuroinflammation, converting a cognitively unimpaired preclinical AD subject to a patient with MCI and finally with dementia. With recent advances in ultra-sensitive quantification of pathological proteins in human plasma samples, levels of phosphorylated Tau protein (pTau181) were used to distinguish plasma samples from AD vs. control subjects (Janelidze et al., 2020; Thijssen et al., 2020). For the first time, both Tau and pTau181 can be measured in plasma to predict brain Tau load and neurodegeneration, for monitoring the efficacy of β- or γ-secretase inhibitors/modulators in clinical trials. Existing β-/γ-secretase inhibitors/modulators can then be tested in sub-cohorts selected by AT(N) biomarker.

One plausible explanation for negative clinical results is that BACE1 inhibitors and GSIs/GSMs were given too late, during the irreversible phase of the disease (MCI/prodromal AD, mild-to-moderate AD), and patients need to be treated at earlier stage of AD to prevent neurodegeneration. The phase III trial of BACE1 inhibitor verubecestat by Merck in patients with prodromal AD (MCI) was discontinued in 2018, similar to the phase II/III trial of atabecestat by Janssen.

In addition, side effects and toxicity account for a number of failed trials, such as the inhibition of Notch signaling by GSIs. Recent studies on BACE1 knockout mice suggested that BACE1 inhibitors may disrupt the axonal organization in the hippocampus, and impair synaptic plasticity, leading to defects in learning and memory (Ou-Yang et al., 2018; Lombardo et al., 2019). Inhibition of γ-secretase cleavage can lead to the accumulation of C99 fragment (Lauritzen et al., 2019a), which was correlated to the acceleration of early neurodegenerative process in AD (Lauritzen et al., 2012; Lauritzen et al., 2019b).

**FIGURE 8:**
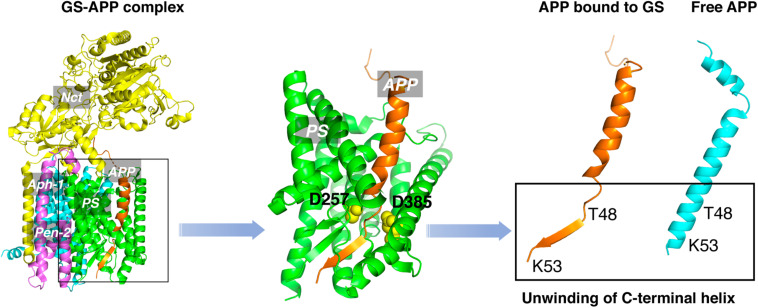
The unwinding model of γ-secretase-APP interaction.

An obstacle for the development of selective GSMs is the lack of information about the structural characteristics of γ-secretase-substrate complex. Recent cryo-EM structure of γ-secretase bound to the C83 fragment and Notch substrate (Yang et al., 2019; Zhou et al., 2019) started to fill this crucial knowledge gap. An α-helical to β-strand transition was observed at the C-terminal of APPTM, forming an anti-parallel, intermolecular β-sheet with two induced β-strands from presenilin (Figure 8). The unwinding of C-terminal transmembrane helix exposes the initial ε-cleavage sites (T48, L49) to interact with γ-secretase (Zhou et al., 2019). These are in agreement with earlier Raman and NMR spectroscopic studies carried out on APPTM and PSHs in solution, which showed that substrate binding is coupled with helical unwinding to prime the substrate for peptide bond hydrolysis (Brown et al., 2018; Clemente et al., 2018).

This unwinding model of the substrate in γ-secretase-APP interaction suggests that targeting APP, especially the C-terminal of APPTM, could be a new strategy in selective amyloid reduction. A substrate-specific inhibitor is not expected to affect the γ-secretase cleavage of other physiological substrates, the assembly of the γ-secretase complex, or any presenilin function (Saura et al., 2004; Wines-Samuelson et al., 2010; Watanabe et al., 2012; Barthet et al., 2013), thereby sparing the side effects associated with broad-spectrum γ-secretase inhibitors. A novel compound C1 was found to interact with the C-terminal juxtamembrane lysines of APP and inhibit the γ-secretase production of Aβ both *in vitro* and in cell (Zhao et al., 2020). This study provides the first *in vitro* evidence that targeting the C-terminal juxtamembrane lysines is sufficient for reducing Aβ production.

Besides cleavage by the well-known α-, β-, and γ-secretases, APP can be alternatively cleaved by meprin-β, legumain, rhomboid-like protein-4 (RHBDL4), caspases, η-secretase (MT-MMPs) at different sites, resulting in different APP fragments (reviewed by García-González et al., 2019). These emerging proteinases in APP metabolism opened more possibilities for the intervention of amyloidogenic APP processing in AD. For example, cleavage of APP by meprin-β can generate a N-terminally truncated Aβ (Aβ_2–X_), which is more prone to aggregation than Aβ40; while the inhibitor of meprin-β, fetuin-A, was reduced in CSF of AD patients (Puchades et al., 2003), indicating a potentially pathogenic role of enhanced meprin-β activity in AD. Further investigations are needed to link the new proteinases to AD pathogenesis, which will offer novel insights and more molecular targets in AD drug discovery.

AD is a complex disease and therefore drugs with a single molecular target may not be sufficient for reversing the progression of AD. Thus, multi-target molecules able to inhibit both APP processing and tau pathology, or neuroinflammation etc., may be a promising approach for the treatment of AD. Emerging multi-targeted molecules are developed based on natural products and their derivatives, such as curcumin, berberine, and epigallocatechin gallate (EGCG) (Shan et al., 2011; Di Martino et al., 2016). Multiple AD-related clinical trials were carried out for EGCG, which is a polyphenolic flavonoid extracted from green tea. EGCG has been proposed to not only inhibit Aβ misfolding and tau aggregation *in vitro* but also increase α-secretase cleavage of APP and improve inflammatory in APP/PS1 transgenic mouse models (Obregon et al., 2006; Ahmed et al., 2017; Guéroux et al., 2017; Ettcheto et al., 2020). While the outcome of clinical trials on EGCG is currently inconclusive, more trials are ongoing and it represents an intriguing multi-target strategy in AD treatment.

## Summary

Drug discovery targeting Aβ-producing enzymes is one of the most important strategies to develop disease modifying therapeutics for AD. Exploration of “Notch-sparing” GSMs and inhibitors targeting the substrate APP are two promising future directions. Beyond obvious significance in AD drug discovery, investigation of the γ-secretase and BACE inhibitors will not only provide chemical probes to study fundamental enzymatic mechanisms, but also help settling the debates on whether Aβ is indeed a molecular driver in AD pathogenesis.

## Author Contributions

JZ and CW conceived and wrote the manuscript. XL, WX, and YZ edited the manuscript. All authors contributed to the article and approved the submitted version.

## Conflict of Interest

The authors declare that the research was conducted in the absence of any commercial or financial relationships that could be construed as a potential conflict of interest.
